# Quarterly Periscope, or, Spirit of the Public Journals

**Published:** 1823-12-01

**Authors:** 


### Quarterly Periscope

OF

### PRACTICAL MEDICINE;

BEING THE

### Spirit of the Public Journals,

FOREIGN AND DOMESTIC.

*Paucis libris immorari et innutriri oportet, si velis aliquid trahere, quod in animo fideliter hæreat.* SENECA.

Duo vitia vitanda sunt in cognitionis et scientiæ studio. ***** Alterum est vitium, quod quidam nimis magnam operam conferunt in res obscuras atque difficiles, easdemque non necessarias. CICERO.

1. *Researches on the Pathological Anatomy of the Digestive Canal.* By M. ANDRAL, Jun. M. D.* The following memoir was read at the *Académie Royale de Medecine*, in the sittings of March and April 1822, and received the highest commendation from Messieurs Chaussier and Clocquet, who were deputed to render some account of it to that learned body. The pathological observations contained in it appear to be possessed of great interest, and seem evidently written by one who must have paid very considerable attention to the anatomy of the body under health as well as under disease.

SECT. I.—*Anatomical characters of inflammation of the digestive tube.* The digestive canal, inflamed, when viewed externally, is generally contracted, and appears injected. Contraction of the intestines, however, sometimes exists independently of inflammation. It is not unfrequent, for example, to find the pyloric portion of the stomach strongly contracted and reduced to the size of a small intestine, although no mark of inflammation can be discovered on its internal surface: we have also frequently seen the great intestine strongly and universally contracted from the cæcum to the rectum, and yet no derangement in its mucous coat. The injection, when seen externally, is sometimes seated in the sub-peritoneal tissue only, more commonly it has its seat in the cellular layer, situated between the mucous and muscular coats, but in no case can the external appearance show the state of the mucous coat. It has frequently happened to us, to find this membrane evidently inflamed, disorganized, and ulcerated, in portions of the intestines, which, when seen and examined externally, had been regarded as healthy. An important error may therefore be committed, if, as is sometimes done, we pretend to judge of the healthy or morbid condition of the intestine by the appearance of its external surface.

If we examine its internal surface, we find it present a multitude of different aspects: an uniform red colour is often observed of a greater or less extent, which varies from the most intense vermilion to the deepest

* Nouveau Journal de Medecine, as in Nos. 111 to 114 of the London Medical Repository. We have deemed it proper to publish the whole of this Memoir at one view; and that it may not be said we did so to save trouble, we have given it entirely at our own private expense *beyond* the regular limits of the Journal. *Ed.*

brown. Sometimes this redness loses itself by little and little, as is observed more particularly in the small intestine; whilst at other times it terminates abruptly, as is frequently observed at the union of the pyloric portion of the stomach with the splenic, at the junction of the stomach and duodenum, and at the ileo-cæcal valve, the superior surface of which is often found very red, whilst the inferior is very white, and *vice versa*. The valvulæ of the small intestine are commonly of a much deeper red than the intervals which separate them; but if they are unfolded, this more intense colour is observed to disappear: at other times there exist, in several places, red or brown patches of a round or irregular figure, between which the internal surface of the intestine is nearly white: these patches seem to form so many isolated phlegmasiæ. It is not uncommon, in place of an uniform red tint, to meet with numerous arborescent appearances, occasioned by a strong injection of the vessels, and their numerous anastomoses; around these vessels a crowd of small red points frequently appear, which seem to be sometimes owing to a more violent partial injection, and occasionally to a slight circumscribed sanguineous extravasation. Finally, pimples, pustules, and fungous excrescences of different forms and sizes, sometimes arise from the internal surface of the intestine.

It is not sufficient, however, to have opened the intestinal canal, and to have observed, with more or less attention, the state of the internal surface: the mucous surface must be separated from the subjacent tissues; must be studied when thus separated; and the lesions which the other tunics have undergone, be afterwards investigated. This is the manner in which we shall proceed at present.

If we open the stomach, or any portion of the intestine of a living animal, we find that, except during digestion, the mucous membrane is every where of a white or rosy white colour; through its tissue, which is perfectly transparent, the subjacent laminated tissue may be discerned: it is commonly but triflingly injected; but it becomes more so if the animal struggles much. During the process of chymification, the mucous coat of the stomach is commonly of a deepish red colour: in proportion as the chyme passes forward in the intestines, their mucous coat becomes vascular like that of the stomach. The same colour is observed in the portions of the great intestine, where the fecal matters accumulate and sojourn. Thus, in several rabbits where we have had occasion to examine the digestive tube, we have found the mucous coat of their enormous cæcum, which is always filled with a great quantity of fæces, of a lively red.

With regard to thickness, the mucous membrane of the digestive passages presents great varieties in its different parts. In the stomach it is at its *maximum;* it is thinner in the small intestines; and still more so in the cæcum and colon, where it only forms a sort of very thin cuticle.

Its consistence is every where in a direct ratio to its thickness: thus, in the stomach it can easily be detached in large flaps, and may be pulled in different directions without tearing, whilst in the great intestine it tears on the least effort.

Its adhesion to the subjacent laminated tissue is by no means considerable in the stomach. In the duodenum it can only be separated with great difficulty: in the remainder of the small intestines, the adhesion of the mucous coat is more intimate where it covers the valvulæ, than in the intervals between them; in the great intestine this adhesion is tolerably strong.

Such has appeared to us the mucous membrane of the digestive canal in its physiological state. When attacked with inflammation, it presents numerous modifications in its colour, thickness, consistence, texture, and frequently in the state of its follicles: the liquids which cover its surface, also undergo remarkable changes with respect to quantity and quality.

The first effect of inflammation is to occasion a greater flow of blood into the part of the mucous coat which is attacked by it: when detached under such circumstances, and held between the eye and the light, this membrane sometimes appears overrun by numerous vascular networks; whilst at other times it presents an uniform red tint, and completely intercepts the passage of the luminous rays. This latter condition indicates a more considerable afflux of blood: the smallest vascular ramifications are filled with it, and are so closely compacted that no interval exists between them. Anatomists, in their artificial injections, can easily give this uniform red tint to the tissues, and especially to those which are membraneous. In place of presenting a red colour, the mucous membrane, when in an inflamed state, is frequently of a *more or less deep brown colour* This tint does not depend upon the duration of the inflammation: it is sometimes observed to supervene in a very short space of time when the inflammation is violent, at which times it seems to announce the commencement of the disorganization of the membrane. Carry into the stomach of an animal some strong corrosive poison, such as corrosive sublimate, arsenic, sugar of lead, &c. and you will find in a very short space of time, an hour for example, the mucous membrane of a cherry red in several parts, whilst in others it is of a brownish grey. Sometimes this latter colour exists alone. Professor Orfila found that the mucous coat of the stomach was of a very deep reddish brown, in animals which had swallowed euphorbium about twenty-four hours previously. In man, the mucous coat of the stomach frequently presents the same tint, whilst at the same time it is softened.

It results from these facts, that the brown colour of the mucous membrane of the digestive canal is a product of its inflammation, and that it may equally manifest itself, in violent inflammations, which occasion, in a very short space of time, the disorganization of the tissue with which they are placed in contact; and in chronic, which in process of time produce the same result.

At the same time that the mucous coat becomes injected with blood, it must be necessarily thickened. This thickening, like the inflammation which gave it birth, may be general or circumscribed. It is particularly well marked in chronic inflammations: we have oftentimes seen, in cases of chronic diarrhœa, the mucous membrane of the colon of a thickness equal to that of the four tunics united, in their ordinary state.

The circumscribed thickening of the mucous coat is tolerably frequent: it appears under the form of round or oblong patches, projecting above the remainder of the internal surface of the canal, to the extent of two or three lines. The surface of these patches is glossy and rugous; the mucous membrane which surrounds them is sometimes perfectly white and transparent, sometimes more or less strongly injected. Their dimensions are variable: the greatest which we have seen, were at least of the size of a crown piece: the smallest were about the size of a shilling. These partial thickenings of the mucous coat are very rare in the stomach; more common in the great intestine, especially in the transverse colon, and more common still in the inferior part of the small intestine. Sometimes one only of a greater or smaller size is met with: at other times they are found to exist in very great numbers. With respect to colour, two species may be admitted: some are red, and appear to be of recent formation. whilst others are of a white, more faded than the rest of the mucous coat, and appear to be the result of an old inflammation, the other signs of which have disappeared: it is a sort of termination by induration.

When the thickening of the mucous tunic is considerable, it preserves, in the greater number of cases, its consistence, or even acquires a greater density; but at other times we find this membrane of its ordinary thickness, and singularly softened.

Pathological observations and experiments made on living animals, show that softening of the mucous coat may take place in a very short time, provided the inflammation be very violent. Half an hour after some grains of corrosive sublimate had been injected into the stomach of a dog, Mr. Brodie found that the mucous coat of the organ had undergone a remarkable softening.

In this condition it is impossible to detach the mucous tunic under the form of membrane; it is demi-liquid, and the slightest rubbing with the scalpel, or friction with the finger, reduces it into a sort of pulp or reddish *bouillie*.

A similar softening is sometimes observed in mucous membranes, which are perfectly white. Must not this condition, like the white circumscribed patches of which we have already spoken, be considered as the sequel of an old inflammation? We shall see farther on that this pulpy sort of condition of the mucous coat, with whiteness of its tissue, is one of the lesions which the great intestine presents, in cases of chronic diarrhœa.

Not only does the mucous coat, when in a state of inflammation, become thickened and softened, but *vegetations* sometimes arise from its internal surface, of a red or brown colour, concave form, and of extreme softness, pressed one against the other, and projecting four or five lines above the rest of the membrane. They somewhat resemble the papillæ of the under surface of the tongue, supposing those divided in small fragments in the direction of their length; and, like them, they are loose and moveable. A great number of similar vegetations were found in the stomach of a man, who died two months after having taken powdered cantharides (Orfila). We have observed them in the large intestine only.

In place of these vegetations, the mucous membrane sometimes presents white conical *pustules*, projecting from half a line to a line, being about as large at the base as a lentil, the majority depressed at the summit, and exactly resembling variolous pustules. They are rarely found isolated, being most commonly grouped, like the eruption of confluent small-pox. Between them the mucous membrane is sometimes red, sometimes scarcely injected. We have never met with these pustules in the stomach. Once we have observed the internal surface of the duodenum covered with them in its upper portion; we have seen them three times in the transverse colon; but it is in the two lower fifths of the small intestine that they most commonly show themselves.

We have more frequently witnessed in the colon pustules of a different aspect. They are conical like those just mentioned, and are situated in the mucous coat, which, when removed, takes them along with it. Their base is, however, much broader, their height more considerable; they terminate in a pointed head; they are of a deep cherry red, and the mucous coat around them is but little injected. We cannot give a more exact idea than by comparing them to those small tumours of the skin, known under the name of furuncles or boils.

M. Lerminier has proposed to designate these different species of eruptions under the generic name of *exanthema internum (exanthème interne.)* This is one of the lesions which the intestines present in fevers, as we shall elsewhere show.

The mucous coat, when inflamed, is modified in its functions, like other tissues, when attacked with inflammation. The examination of the changes which the constant absorption undergoes, that takes place at its surface, does not belong to the province of pathological anatomy; we shall not, therefore, enter into it here, but proceed to point out the modifications experienced by the liquids secreted by it, both as regards their quality and their quantity.

The changes in the quality of the *gastric and intestinal mucus* cannot always be easily appreciated, in consequence of its either being mixed with the drink and chyme, or with the biliary and pancreatic fluids.

It presents two important modifications: sometimes, instead of being viscid, ropy, tolerably consistent, it becomes much more liquid, and resembles serum; at other times, on the contrary, it becomes more plastic, concretes, and is transformed into a *false membrane.* All authors have spoken of false membranes lining the internal surface of the colon in dysentery, and being evacuated per anum. May they not, in some cases, become organized, adhere together like the false membranes of the serous coats, and occasion the obliteration of a part of the intestine? We read, in truth, in authors, of examples of inflamed intestines which were by that means partially obliterated. We have never had an opportunity of witnessing the organization of this species of false membranes. We have never observed these membranes except in the stomach and great intestine.

We have found the whole of the internal surface of the pyloric portion of the stomach lined by a greyish, tenacious layer, which might be torn into flaps, and simply lying on the mucous coat, of which it was, at least, the thickness. In a young girl, twelve years of age, the internal surface of whose stomach presented a great number of red patches, extending in the form of broad stripes from the cardia to the pylorus, we found each of these patches covered by a membraniform, greyish, albuminous layer: between them the internal surface of the stomach, which was very white, was not covered with any false membrane.

It is not uncommon to find the portions of an inflamed intestine filled with a *reddish liquid,* which appears to be owing to a mixture of mucus and blood. The cæcum and the end of the small intestine are the parts where it is most commonly met with. We have sometimes observed bundles of ascarides situated in this bloody liquid, which existed in no place except surrounding them.

On injecting corrosive sublimate into the subcutaneous cellular tissue of an animal, Dr. Smith found the mucous coat of the stomach covered with an abundant *sanguineous exhalation.*

The *quantity of liquid* exhaled by the mucous coat, in a state of inflammation, is sometimes prodigious. Morgagni has cited the example of a woman, who, in one day only, passed, *per anum,* forty pounds of a limpid fluid. The debility into which such a considerable discharge throws the animal economy, may be sufficiently considerable to occasion death in a very short space of time.

It is not the secretion of the mucous coat only which in this case is modified and augmented. Dissection demonstrates that the inflamed intestine solicits a more abundant *secretion of bile,* which flows towards the parts labouring under inflammation. Stoll has noticed this fact: but far from regarding the afflux of bile as being owing to a pre-existent inflammation of the intestine, he regarded the latter as an effect of the presence of the hepatic fluid.

The *sub-mucous cellular tissue,* in the greatest number of cases, remains untouched beneath the mucous coat, when the latter is violently inflamed and even disorganized. Occasionally, however, it is found pretty strongly injected; sometimes, also, like the cellular tissue which surrounds the arteries, it acquires an extreme fragility. We may then very easily detach the mucous coat without tearing it, to the extent of several inches: in some cases sanguineous effusion takes place between its interstices, (*dans ses mailles*,) some examples of which we have met with in the human subject. M. Orfila observed it in animals poisoned by the daphne laureola. Finally, in many chronic inflammations, the sub-mucous cellular tissue becomes more dense, and of a scirrhous hardness; at such times, its thickness exceeds that of all the tunics united. More rarely we have found it softened and of a pulpy nature.

The *muscular coat* presents few alterations in its texture. We have, however, seen it sometimes very much softened, the slightest traction at such times being sufficient to tear it. In other cases it has appeared to us considerably thickened. We have found it in the transverse and descending colon of a scirrhous density and hardness; but the disposition of its fibres was still very distinct, the longitudinal and circular arrangement being perfectly perceptible. Finally, we have sometimes met with it of a deep red, whilst in its sound state it is white.

But if inflammation of the mucous coat is rarely propagated to the muscular, we frequently observe the latter sympathetically irritated, and contracted in parts where the stimulus is applied. This fact may be readily proved by direct experiments: if we inject an irritating substance into any portion of the intestinal canal of an animal, the intestine is observed to become indurated and to contract, at the part where its internal surface is in contact with the poison. The contraction of the intestine continues after death, as we have already seen: being commonly partial, like the inflammation, it very rarely exists in the whole extent of the canal. One of the most remarkable facts of this kind is related by M. Tartra in his thesis:—An individual died, three months after having swallowed nitric acid; the digestive canal was reduced to so small a volume, *that it might* (as it were) *have been held in the hollow of the hand,* its calibre, through the whole length, not exceeding, in size, the barrel of a quill.

May not the irregular and multiplied contractions, of which the muscular membrane of the inflamed intestine becomes the seat, be regarded as one of the causes of *invaginations of the intestines?* Boyer has well described the mechanism of these invaginations, from observation on living frogs. By irritating the intestines of these animals, he saw them contract forcibly in several parts, whilst in others they remained distended with aliment and flatus; these latter portions he saw receive the former. "Hæ ità ampliatæ intrà se receperunt constrictas intestini portiones, easque sinu suo absconditas aliquam diù detinuerunt, donec fibris se denuò exporrigentibus intestini pars una è latibulo alterius, velut è domunculo limax, pristinam in sedem rediret."

But although several authors have remarked that they have constantly found the mucous coat very much inflamed at the part where *intus-susceptions* existed, and have regarded them either as the cause or the effect of such inflammation, we can affirm that they more frequently exist without inflammation of the intestines. It is probable that many of them are found only at the moment of death, a period at which the peristaltic motion of the intestines becomes much more active, as experiments, made on living animals, demonstrate.

Considered with relation to their seat, intestinal intus-susceptions are far from being equally frequent in the different portions of the digestive tube. The ileum is the most common situation in which they occur. Fabricius Hildanus and Bartholin have seen the cæcum received into the ileum; Hartmann, on the contrary, found the ileum received into the cæcum. Invaginations of the colon are very rare. Meckel says he has seen the transverse and descending colon introduced into its sigmoid flexure. Bonetus has related a case of invagination of the rectum in a man who died of obstinate obstruction of the bowels, accompanied with vomiting of a fæcal matter. Can we believe the assertion of a German writer, who says he has seen the duodenum invaginated in the ductus choledochus?

With regard to their disposition, the superior portion of the intestine is most commonly received into the inferior. Sometimes they are very numerous: we have observed as many as seven in the same subject. Their length may vary from some inches to upwards of two feet.

The symptoms occasioned by invagination of the intestines vary according to their size, situation, number, and degree of obliteration of the intestine.

The *subperitoneal cellular tissue* is still more rarely affected than the sub-mucous: like the latter, it may become very fragile; like it, also, it may acquire considerable thickness. In the latter case, the peritoneal covering is found to be separated from the muscular coat by a cellular layer, two or three lines in thickness, whilst, in the ordinary state, we rather suppose it, as it cannot really be seen.

The *peritoneal coat* almost always remains untouched: occasionally, however, like the two laminated membranes, *(commes les deux membranes lamineuses)* it is very fragile. We have found, in some cases, all the coats of the intestine equally softened at the same time, and to a very high degree. The slightest traction was sufficient at such times to tear its parietes; and when gently rubbed between the fingers, they became reduced into pulp.

This general softening has seemed to us to be more common in the stomach than in any other portion of the intestine.

The mucous coat of the digestive canal, disorganized by inflammation, is apt to become destroyed to a greater or less extent, and whether it is, that the particles composing its tissues are absorbed, and that they are carried away along with the contents of the intestine, *ulcerations* are the consequence, which always originate in the mucous membrane, primitively or secondarily inflamed.

*Ulcerations* may occur in all parts of the intestine from the cardia to the anus; but they are by no means of equal frequency in every portion. Thus they are sufficiently rare in the stomach, and still more uncommon in the duodenum and jejunum. They are very common in the lower third of the small intestine, and less so in the different portions of the great intestine. We may judge of their respective frequency by the following table, which was formed from the results of fifty-three dissections, several of which had ulcerations, at the same time, in many parts of the digestive tube:—

| Seat of the Ulcerations. | | Number of Subjects. |
|---|---|---|
| Stomach | | 9 |
| Duodenum | | 1 |
| Jejunum | | 9 |
| Ileum (lower portion) | | 26 |
| Cæcum | | 10 |
| Colon | Ascending | 4 |
| | Transverse | 11 |
| | Descending | 3 |
| Rectum | | 1 |

These ulcerations are commonly numerous, except in the stomach, where we generally observe only one or two. In the superior portion of the small intestine, they are constantly separated by great intervals. In its inferior portion, we always find them much nearer to each other: they are frequently united and confounded near the ileo-cæcal valve, so as to form, by their union, one large ulcer. In the cæcum it is very unusual to find them so confluent, as well as in the remainder of the large intestine, where the spaces which separate them are commonly more considerable than the spaces, even, which they occupy.

They frequently arise in the middle of the red circumscribed patches of which we have already spoken, and at such times the mucous coat which surrounds them remains white, as around the patches. It is important to remark this disposition, because the perfect whiteness which exists around many ulcerations, had occasioned it to be admitted that they might occur in the mucous membrane without previous inflammation; but it is clear that they are, in this case, the result of partial inflammation.

Ulcerations also follow the pimples and pustules with which the mucous membrane is sometimes covered. If, in fact, we observe attentively these different exanthemata, a slight erosion may be perceived at the tops of many: the summit of some others will be found to have undergone a more considerable loss of substance: the small ulcer which results extends progressively from the top of the pustule to its base, finishing by entirely destroying it. Does not the greatest analogy exist between the mode of the production of these ulcerations, and the development of certain ulcers of the mouth, which likewise owe their origin to small pustules known under the name of aphthæ?

Sometimes, also, the ulcerations are, as we shall hereafter establish, the result of the separation of eschars on the mucous membrane.

Is it in the mucous follicles that ulcerations most commonly commence? Several authors have thought so. The greater vital activity in the follicles than in the other parts of the mucous coat, the more considerable vessels which they receive, the frequently extraordinary secretion of which they become the seat in many intestinal phlegmasiæ, may lead us to suppose, that whenever the mucous coat is inflamed, they are most especially irritated, become disorganized, destroyed, and ulcerated: but no fact shows that this is *actually* the case. The greater frequency of ulcerations in the lower portion of the small intestine is, in truth, in a direct ratio with the greater number of follicles in this same part; but in the duodenum, the follicles are likewise extremely numerous; they are larger, more apparent than any where else, and, notwithstanding this, the duodenum is the part of the *digestive* tube where ulcerations most rarely show themselves.

Nothing fixed can be established regarding the period at which the mucous membrane, when inflamed, becomes ulcerated: it frequently happens that no evidence of *solution of continuity* is to be met with, although it has been the seat of very old and tolerably intense inflammation. The facility and rapidity of its ulceration appear connected with some individual, inexplicable, disposition. This is demonstrated by experiments made on living animals of the same age and strength, which have been poisoned by an equal dose of corrosive sublimate. At the end of forty-eight hours, we find, in some, only a lively red colour of the mucous coat of the stomach, whilst, in others, the internal surface of the stomach already presents one or more ulcerations. We read in Morgagni the case of an individual who, whilst in perfect health, was suddenly seized with most excruciating pain at the epigastrium, and with every other symptom of gastritis. He died at the end of twenty-four hours: ulcerations existed in his stomach.

The size of intestinal ulcerations is infinitely varied: some are so small they will scarcely admit the head of an ordinary pin; others are several inches in diameter in every direction: we have sometimes seen them occupy the whole circumference of a portion of the intestine.

We have found the mucous coat entirely removed for more than six fingers' breath above the cæcum: it is in this part of the intestine and in the stomach that we have met with the largest ulcerations. Some are oblong, and have their largest diameter in the direction of the length, or of the breadth of the intestine; others are exactly circular, and others again are linear.

Their edges are always formed by the mucous membrane. Sometimes they are red, thick, and elevated two or three lines above the base of the ulcer, whilst, at others, they are white, thin, and on a level with the bottom. Occasionally they are irregularly formed, and present numerous fringes, which advance from the circumference towards the centre of the ulcer.

We have often met with the mucous membrane separated to a pretty considerable extent around the ulcers: when they are in great number, and in proximity with each other, the mucous coat which separates them is sometimes entirely, or almost entirely detached from the subjacent tissue. This separation, owing to the morbid alteration of the cellular tissue, is similar to that which is observed around many cutaneous ulcers.

The base of the ulcerations differs according to the time at which we examine it. If the solution of continuity be recent, the laminated tissue which forms its base is thin and white as in its natural state:—it may preserve this appearance for a longer or shorter time: but when the ulceration has already existed for some time, it commonly acquires a considerable thickness, which may be easily felt on touching the external surface of the intestine—it becomes rugous, unequal, granulated—it is of a grey, red, or brown colour—it secretes a liquid which shows only these different tints, which sometims concretes into a false membrane, and forms, at its surface, a layer more or less dense—in some circumstances it assumes a totally black colour, and seems to be transformed into a true eschar. But most commonly the laminated tissue becomes insensibly destroyed, after the manner of parts affected with that species of inflammation which Hunter designated under the name of ulcerative inflammation, and the base of the ulceration is then formed by the muscular membrane. This latter coat sometimes preserves its natural appearance; at others, it becomes progressively very red, soft, black, and destroyed in its turn, leaving the peritoneal membrane exposed. The base of the vast ulcerations at the end of the small intestine, or in the cæcum, frequently show these different coats exposed in different parts of its extent. In some cases, we may in a manner follow with the eye the successive destruction of the coats from the internal towards the external surface of the intestine, and from the centre of the ulceration towards its circumference. At such times, the edges of the ulcers are observed to present, as it were, different stages. The first, the most remote from the centre, is formed by the thin or thickened mucous coat, of a red or white colour; the second, situated more internally, is formed by the laminated tissue; the third, still nearer the centre, is formed of the muscular coat; and, finally, at the base, the thin and transparent peritoneal coat is observable. This last membrane grows altered in its turn: it becomes more fragile, tears, and perforation of the intestine is the result.

Such is the progressive march pursued by these ulcerations, when they extend in depth, but most commonly they seem to have a tendency to extend in width only, at the expense of the mucous membrane alone. Thus, in the majority of cases, we have found the base occupied by the laminated tissue.

As the ulcerations may form very rapidly, so may they extend in depth with a rapidity sometimes terrific. This is attested by various cases of poisoning, in which the intestines have been found perforated in a very short time.

Under some circumstances, the perforation is effectuated from the exterior to the interior. This occasionally occurs in cases where tubercles are formed in the peritoneal coat: becoming soft, this accidental tissue destroys the serous coat, and an ulcer results, the base of which is formed by the muscular tunic. Sooner or later this becomes destroyed likewise, and the parietes of the intestines are formed, under such circumstances, at these parts of the thin and transparent mucous membrane alone: finally, this last gives way in its turn. We have recently witnessed these different stages in a young man affected with tubercular peritonitis *(péritonite tuberculeuse.)*

Intestinal perforations may likewise supervene without previous ulceration, in cases where all the coats which constitute the parietes of the intestine are softened at the same time. The least force exercised upon these parietes by solid, liquid, or gaseous bodies, is, under such circumstances, sufficient to occasion their rupture. Can an intestine, the parietes of which are neither ulcerated nor softened, be so much distended with flatus or liquids as to burst? The stomach of herbivorous animals presents this phenomenon. Frank cites facts which appear to demonstrate that a similar rupture may be produced in man by the accumulation of flatus in a circumscribed portion of the intestines. It is thus that, according to Stoll, the bladder may burst when it is distended by a too great quantity of urine.

May not intestinal worms, in some cases, destroy and pierce the parietes of the canal which they inhabit? or, in cases where many of these animals have been found in the peritoneum, have they passed through a perforation which had been already made? Some observations of Wepfer tend to cause us to admit the possibility of the perforation of the intestine by worms. In the thirteenth chapter of his treatise on the *Cicuta Aquatica*, he observes that, on dissecting dogs, he found lumbrici in their intestines, many of which were still living, and adhering strongly to the parietes of the intestine by one of their extremities, in the manner of a leech: "Proboscis firmissimè internæ intestinorum tunicæ infigebatur, à quâ etiam, sublato intestino, hirudinum instar, pendebant. Hi monstrarunt rationem et modam quo intestina, umbilicum, et inguina perforent."

Whatever may have been the mechanical or vital cause under the influence of which the intestine has been perforated, its cavity communicates either with the exterior—with some viscus—or with the cavity of the peritoneum. Hence arises the very great difference between the symptoms, which follow the perforation and which announce it.

1. *Communication between the digestive tube and the exterior.*—Examples of this are numerous: it is the artificial anus. Adhesions are established between the edge of the perforated intestine and the abdominal parietes, and no effusion can take place into the peritoneum. We can thus explain how a ball may traverse the abdominal parietes, and be voided by stool, without having been attended with fatal effects, *(Ephem. Nat. Cur.)* how a knife swallowed was discharged, at the end of seven weeks, through an abscess formed at the epigastrium, without the patient succumbing. The following case we observed at *La Charité*:—A man, thirty-four years of age, had been affected with diarrhœa for several months; a hectic fever insensibly consumed him; he complained of a violent pain in the right iliac region; this part soon grew tumefied, and fluctuation became manifest. A lancet was passed into the centre of the tumour; this gave issue to some fetid gas, with a few drops of a greyish liquid equally fetid. The following days a larger quantity was discharged; the patient soon fell into the last stage of marasmus. We found beneath the skin of the iliac fossa a vast abscess, containing a greyish liquid of a gangrenous fetor, and circumscribed on every side by thick cellular bands (*brides.*) Scarcely any traces of the muscular fibres of the parietes of the abdomen were recognizable; they were blackish, soft, and, as it were, diffluent. At the bottom of the abscess was the cæcum, the external parietes of which presented a perforation with irregular edges, sufficiently large to admit the extremity of the little finger. Numerous ulcerations existed in the intestine.

2. *Communication between the digestive tube and another organ.*—In this case, as in the preceding, adhesions, established by the beneficence of Nature, prevent any effusion into the peritoneum. Sometimes the intestine opens into a hollow part, into which it empties itself: thus, Frank has seen a communication established between the arch of the colon and the bladder. Sometimes the dense and solid tissue of a viscus supplies the destroyed parietes of the stomach or intestines. Thus we have often seen the bottom of carcinomatous ulcers of the stomach formed by the liver or the pancreas. Frank relates cases in which the parietes of the intestine was supplied by the spleen and by the kidney.

Authors worthy of credit even assure us that they have seen communications formed between the stomach and thoracic cavity. Willis, quoted by Bonetus, found in a subject the diaphragm destroyed, and the stomach opening, by a large aperture at its superior edge, into a pleuritic pouch of the right side. Van Swieten relates a nearly similar example.

3. *Communication between the digestive tube and the peritoneal cavity.*—In this case, the matters discharged from the perforated intestine are effused into the serous membrane. The peritonitis, which is the consequence, is attended with great variety, as regards its march, its duration, and its symptoms.

Most commonly it has a very acute progress; the patients sometimes dying a few hours after the first symptoms have manifested themselves. Some patients are warned by a very particular sensation at the precise moment at which the effusion takes place. A man, whose cancerous stomach gave way during violent efforts of vomiting, informed us that he had distinctly felt as if a ball had descended from the epigastrium towards the umbilicus, immediately before the pains of peritonitis declared themselves. Stoll relates the case of a patient who, when labouring under retention of urine, felt something suddenly give way in his abdomen, and became at the same time stiff and livid: it was the bladder which had given way. The patients experience excruciating pain; they are in a state of extreme anxiety; the features of the face become rapidly discomposed; the pulse is irregular, thready, &c.

This *ensemble* of frightful symptoms is seen to manifest itself suddenly in individuals who, some minutes before, appeared to enjoy perfect health: their digestive tube has been perforated.

These facts prove with what astonishing rapidity ulcerated inflammation may be propagated: through it the slightest enteritis or gastritis may suddenly become mortal. The following case presents a striking example of it:—

A brushmaker, aged seventeen years and a half, of a lymphatico-sanguine temperament, had always experienced very good health. On the 13th of October, 1822, at seven o'clock in the evening, he felt, without any known cause, stupor and general indisposition. During the whole of the night his skin was of a burning heat. The next day, 14th, same state; anorexia, only one stool; abundant perspiration during the night. 15th, he was received into *La Charité*. He perspired again in the nights of the 15th and 16th. At the visit of the 16th, he presented the following symptoms :—Face red ; eyes sparkling ; pains in the limbs; tongue covered with a thick yellowish coat; lips red ; *fœtor oris*—anorexia; slight thirst; belly soft and without pain; no stool for twenty-four hours; pulse frequent, tolerably full; skin moist. (*Barley-water with gum, a lavement of marsh-mallows.*) The patient had only one dejection until the next morning.

17th. Six grains of ipecacuanha were administered; the patient vomited twice a tolerably large quantity of bile ; no motion; in the night slept well; awoke in a slight perspiration.

The next day, in the morning, (18th,) the yellowish coating of the tongue had disappeared, and it was of a fine vermillion colour; the bad taste in the mouth no longer existed ; the pulse was a little frequent; the temperature of the skin nearly natural.

From the 19th to the 22d a slight febrile movement continued : anorexia; same state of tongue. One motion each day after the *lavement*. (*Emollient tissanes; broth.*)

23d. The tongue was much redder ; the frequency of pulse had considerably augmented; the skin was burning; the abdomen painful on pressure. Two liquid motions in the last twenty-four hours. This return of the symptoms was combated by the application of eight leeches to the anus. (*Barley-water diet.*)

During the day, the pains of the abdomen assumed a terrifying intensity, and in the night he began to vomit a great quantity of green, porraceous bile.

In the morning of the 24th we found him lying on his right side: the eye dim ; the face pale and cadaverous; the slightest pressure upon the abdomen, the least movement, occasioned the most violent pain. Continual nausea tormented him, which was followed, from time to time, with the expulsion of some gulps of bile. Respiration quick and laborious; pulse very frequent, small; skin without heat. The existence of peritonitis did not admit of doubt. M. Lerminier presumed that the cause might be referred to an intestinal perforation. (*Forty leeches to the abdomen ; oily fomentations ; mild sinapisms to the legs in the evening. Linseed tea.*)

The vomiting continued to take place during the day.

25th. Eight o'clock, A. M. abdomen less sensible, but swollen and renitent; no fluctuation could be felt; the limbs were cold; the pulse thready; the eye, however, had still a tolerably natural expression: the intellect was clear; the speech free. (*Blisters to the thighs.*)

He died at 5 o'clock in the evening.

*Sectio Cadaveris, performed fifteen hours after death.*—Albuminous flakes, forming false membranes, united together the folds of the small intestine. A turbid, lactescent, very fetid, serous fluid was effused into the two flanks and into the hollow of the pelvis. The peritoneum underneath the albuminous flakes was strongly injected. The mucous membrane of the stomach was every where white and sound : an equal degree of whiteness was observable in the whole extent of the small intestine; but about a foot above the ileo cæcal valve there existed five or six oval pimples, as white as the mucous coat surrounding them. The centre of one of these was ulcerated : the base of this ulceration, formed by the serous membrane, had in its centre a round perforation of a line and a half or two lines in diameter. Round these pimples the mucous membrane was covered with several small, white, miliary pustules, developed in its interior, and scarcely projecting above its surface.

The great intestine was perfectly sound, as well as the other viscera. A tubercular mass, of the size of a small nut, existed at the top of the right lung. The exacerbation of the 23d correctly marked the period at which the ulceration took place: the perforation was produced a few hours afterwards.

When a perforation of the intestines supervenes in an individual labouring under typhus fever, with prostration of strength, and remarkable diminution of the general sensibility, it is not always easy to detect the peritonitis occasioned by it. In cases of this kind, in fact, we have seen the patient exhibit no signs of pain, even when the abdomen was strongly pressed upon, in any and every part. The sudden alteration of the features, the unusual tension of the belly, the change in the pulse, which becomes suddenly small and hard, may, at such times, cause us to suspect the existence of inflammation of the peritoneum: acute peritonitis, without pain, supervening on perforation of the stomach and occurring in fever, has been noticed by Morgagni.

Finally, we have seen peritonitis, occasioned by a solution of continuity in the parietes of the intestines, observe a chronic progress. A young man labouring under phthisis pulmonalis had had a copious diarrhœa for a long time: the abdomen had always been without pain. One day he complained of violent pain round the umbilicus; pressure increased it: it was regarded as the product of the inflammation, of which the digestive tube was the seat: this continued incessantly, but not severely, during eight or ten days; none of the other symptoms became aggravated in a remarkable manner: the patient suddenly felt his abdomen wetted by a tolerably large quantity of liquid, and perceived that a linear crack existed at the umbilicus: during the day an ascaris lumbricoides was discharged, along with a yellow liquid similar to that commonly contained in the small intestines. Was it not reasonable to suppose that a portion of the intestine had been perforated; that by the aid of partial adhesions contracted between it and the parietes of the abdomen, no effusion could take place into the peritoneum; and that the abdominal parietes had, in their turn, become inflamed and perforated? Was it not, in a word, a preternatural anus which had been formed? On the following days, however, a small quantity of liquid continued to be dischaged by the fistula; the abdominal pain was somewhat intense. The patient, who had arrived at the last stage of pulmonary consumption, succumbed twenty-seven days after the first appearance of pain, and about eighteen days after the formation of the fistula. The traces of a dreadful peritonitis were discovered. The intestines were united into one mass by black and very thick false membranes: a quantity of greenish liquid was effused between the folds of the intestines; it was there retained by membranous bands, which formed the parietes of a number of small cavities: no adhesion existed at the umbilical region: in the peritoneum two ascarides lumbricoides were found; their presence did not permit a doubt regarding the existence of an intestinal perforation, but the adhesions were so multiplied and so intimate, that it was impossible to discover it.

Stoll has related the case of a young man, who, after having laboured for six months under frequent vomiting and diarrhœa, was seized with violent pain in the abdomen after having taken cold: the twelve following days, he walked a considerable distance, each morning, in order to receive his medicine at the hospital; on the twelfth day he proceeded on foot as usual. The abdomen was tense and painful to the touch, countenance anxious and emaciated, pulse very frequent and small. He died in the evening, a short time after having himself given the preceding details. The peritoneum contained a bloody fluid, mixed with liquid stercoraceous matter. The ileum, not far from the cæcum, presented a hole sufficiently large to admit a filbert. The whole of the digestive tube was, moreover, inflamed.

There are some fortunate cases in which perforation of the intestine has not been followed by effusion into the peritoneum. Thus we have seen one of these perforations comprised between the two laminæ of the mesentery, out of the cavity of the serous membrane. We have also seen the spleen exactly applied upon the perforated stomach at its great *cul-de-sac*, without adhering to it, prevent any effusion. Professor Chaussier has observed some facts of this kind. Can we believe that, except in these rare cases, perforations of the digestive tube may take place during life, without consequent peritonitis? The anatomists who say they have observed this fact, may they not themselves have occasioned the perforation by pulling the intestinal tunics previously softened or deeply ulcerated?

Inflammation of the intestines may, like that of other parts, determine by suppuration and gangrene.

*Suppuration* is commonly established at the free surface of the mucous membrane; but in some more rare cases it is beneath it, in the meshes of the laminated tissue, that pus is formed, collects in *foyers*, and constitutes a sub-mucous abscess similar to the abscesses which frequently form in the tonsils. Similar purulent collections are extremely rare in the sub-diaphragmatic portion of the digestive tube. We have witnessed an example in the duodenum: at two fingers' breadth below the pylorus, a tumour projecting into the interior of the intestine, but not visible at the exterior, appeared; it was soft, fluctuating, and of the size and form of a cherry: the mucous coat, raised up and separated at this part, had preserved its white colour. On cutting into the tumour, a white inodorous liquid, of the consistence of cream, was discharged, which presented all the qualities of healthy pus. It was collected in the laminated tissue, the laminæ of which it had separated in such a manner, that the muscular membrane lay exposed below the collection.

We have several times observed in the lowest quarter of the ileum, beneath the mucous coat, when in a state of inflammation, small white maculæ of the size of a lentil, formed by a pearl-coloured liquid; changing its situation, and spreading *en nappe* when the mucous coat was pressed upon. It appeared as if some pus had been effused into the sub-mucous cellular tissue, which had occasioned the formation of a crowd of small *foyers*, in every part where the inflammation had been the most acute, or where the cellular tissue had yielded the most to distention. It is not uncommon to find, in the sub-arachnoidean cellular tissue, purulent collections, appearing like those of which we have just spoken, under the form of small white isolated maculæ, and, like them, capable of being displaced by pressure.

Authors have spoken much of *abscesses*, formed in the *stomach*, and having been discharged by vomiting. I have never witnessed a case of this kind.

*Gangrene of the intestines*, also, seems to us less common than has been for a long time imagined. Open the different works in which dissections are related, and you will find that their authors speak incessantly of *intestines gangrenous* and sphacelated in a great part of their extent; but unfortunately no one amongst them has described the lesion which he regarded as constituting a gangrenous condition. It is, at least, doubtful whether the brown colour of a more or less extensive portion of the mucous membrane, with softening of its tissue, should be considered as a sign of mortification. Of these derangements of the mucous coat we have already spoken.

There are diseases, as typhus fever, however, in which it is not uncommon to find on the internal surface of the digestive canal, real eschars, similar to those of the skin. Sometimes these eschars are formed at the expense of the laminated tissue which constitutes the face of the ulcerations: at such times it is found thickened, black, or rather of a dirty greyish colour, and of a putrescent consistence, like the *detritus* which exists at the surface of wounds struck with hospital gangrene.

Sometimes the mucous membrane itself, when inflamed, terminates in gangrene. An ulcer succeeds to the falling off of the eschar which results from it. It has appeared to us, that gangrene has tended to develope itself more frequently in the centre of the circumscribed and elevated patches of which we have spoken above. We have sometimes been able to trace, in the same subject, the formation of the eschars in the different *phases*. We observe, for example, at the end of the small intestine, oblong elevations of a brownish red, formed by the thickened mucous coat. In other places, a part of these elevations is changed into a hard tissue of a yellowish brown colour; repeated washing and long maceration cannot efface this tint. All those who have seen this morbid appearance, have not hesitated to compare it to the eschars, with which the surface of blisters is sometimes covered. The gangrened portion, moreover, is already partly detached; it no longer shows itself except in isolated points, in the interval of which ulcerations exist; the base of these is sometimes white, sometimes of a deep brownish red, according as the cellular tissue which forms it has, or has not, participated in the inflammation. In some ulcerations, the eschar, detached in one portion, only adheres to their edge by a thin pedicle; other ulcerations no longer present any trace of it. We have only once witnessed one of these eschars in the stomach: the end of the small intestine and the cæcum are their most common seat. Almost every time we have met with them, their formation has seemed to have coincided during life with the mortification of blisters. Were it not wandering from our subject, we might deduce some important considerations from these facts, both on the nature of the disease, and on the mode of treatment proper for it. These we shall make the subject of another work.

We have now passed in review the anatomical characters of inflammation of the digestive tube; the lesions which it presents appeared to us sufficiently distinct, to enable us to establish three stages of this inflammation, in the same manner as M. Laennec has established three stages in pneumonia, according to the different states of the lung. In the first stage there is simply a more or less strong injection of the mucous coat. The second stage is marked by alteration of its texture, whether it be thickened, softened, or exanthematous. This alteration may, or may not, extend to the other tunics. In the third stage the mucous and subjacent tissues become disorganized and ulcerated.

The second and third stages can never be mistaken; but not so with the first. In the same manner as sero-sanguineous *engorgement* of the lungs, supervening, only, during the latter periods of life, may be easily confounded with the first stage of pneumonia; so may the mechanical *stasis* of the blood in the mucous coat of the digestive organs, or beneath it, be mistaken for an inflammation of these parts. We shall endeavour to offer some considerations which may prevent us from committing a similar error. Whenever, some hours previous to death, the return of the venous blood towards the right cavities of the heart, has experienced a considerable interruption, the parietes of the intestinal canal are found more or less strongly injected in several parts. The obstacle to the return of the blood towards the heart may be seated either in the heart itself or in the lungs. In each case, the blood, which no longer penetrates these two organs but with difficulty, reflows towards the liver, which becomes engorged in its turn, and is no longer able to admit that which is brought to it by the vena portæ. The ramifications of this vein therefore remain filled; they receive at the same time a fresh quantity by the arteries until death, and sometimes even after death. Hence proceeds the injection of the parietes of the intestinal canal; this injection is more lively and more common than that of any other part, either in consequence of the number and size of the vessels, or owing to the presence of the liver, into which the greatest part of the blood received by the right side of the heart, even that which is brought to it by the vena cava superior, reflows and accumulates as in a reservoir. If, in fact, as we have seen done by M. Majendie, we inject sulphuric acid into the jugular vein of a living animal, the hepatic vessels are found filled with coagulated blood. It is only when the engorgement of the right side of the heart and of the liver has reached a very high degree, that the other parts are found also injected with blood after death. At such times the skin is marbled with livid streaks; the cerebral membranes are of an intense red; the brain itself is covered with an infinite number of small red points; an enormous quantity of blood streams out from all the parenchymatous tissues; the inter-muscular, sub-serous, and sub-arterial, cellular tissues, are overrun by a multitude of small vascular ramifications, &c.

But the purely mechanical injection of the intestinal parietes itself presents several degrees.

In its mildest degree, the laminated tissue underneath the mucous coat is found overrun by large veins filled with black blood: they give to the stomach, when viewed internally, a marbled appearance: in the intestines they form, by their multiplied anastomoses, numerous arborisations; they exist, in great quantities, in the folds of the small intestines, which, being deeply situated in the hollow of the small pelvis, present, owing to their inclined position, a fresh obstacle to the return of the blood.

This first degree, which consists in an injection of the great vessels of the sub-mucous tissue, must not be confounded with the inflammatory injection which is seated in the vessels of the mucous membrane.

But in a second degree, besides these veins gorged with blood, the laminated tissue presents a crowd of small vascular ramifications which extend in several parts to the mucous coat: at such times this membrane presents, at greater or less intervals, patches of a brownish red, formed by the agglomeration of several almost capillary vessels, strongly injected. When less numerous, they form, in the mucous coat, small red isolated or united points: when more multiplied, they produce long, red, or brown bands.

Sometimes, sanguineous infiltration, true ecchymoses, with or without injection of the mucous coat, are discovered in the laminated membrane.

Finally, in the highest degree of this mechanical injection, the mucous coat exhales blood at its free surface: this we have witnessed in several subjects. Boërhaave had already observed that when the blood which returns from the intestines cannot pass through the vena portæ, owing to its being obstructed, it flows into the intestines.

This sanguineous exhalation, joined with the interruption of the circulation, is equally observed in other parts. The tissue of the lung, the cerebral substance, the parts of the skin deprived of epidermis, become sometimes its seat in individuals labouring under aneurism of the heart. The bronchiæ are often filled with a bloody fluid during the last moments of the life of consumptive individuals, who have experienced a long agony.

These phenomena, exhibited to us on the inspection of the dead body, experience may produce on living animals. By slowly depriving them of life, we see their digestive tube, which is pale or of a rosy white in the ordinary state, becomes injected and strongly reddened. An intense coloration of the intestines may be also obtained by tying the trunk of the vena portæ. This fact was known in the time of Morgagni: he relates, that after the ligature of this vein, the intestines quickly acquire the colour of cochineal, and that a sanguineous exhalation sometimes takes place at their internal surface.

From the whole of the preceding facts, it results that, when any stimulus affecting the intestines has only occasioned their injection, without having yet altered their texture, it is often difficult, and sometimes impossible, to distinguish this inflammatory injection from one which has been purely mechanical. It is necessary, therefore, to attend either to the symptoms which preceded death, or even to the kind of death; to observe the state of the lungs, of the right heart, of the liver, and of the system of the vena portæ: finally, in some cases we must be compelled still to remain in doubt.

Section II. *Adventitious Tissues.—Tubercles.*—We seek in vain, in ancient authors, for an exact description of the tubercles which develop themselves in the parietes of the digestive tube. They have all, at most, been indicated in a vague manner by some. Thus, Bartholin says he found the intestine full of purulent tubercles in an individual labouring under chronic dysentery. Bonet appears to have spoken of them under the name of apostemata. Brunner seems to have regarded them as mucous follicles preternaturally developed. It is somewhat astonishing that they should not have fixed the attention of Morgagni.

Intestinal tubercles are, in fact, one of the most common affections. They exist in the majority of individuals labouring under phthisis pulmonalis. In the majority of cases they only supervene consecutively on the tuberculous degeneration of the lungs: at other times they precede it, and drag the patient to the grave, before any symptom has declared itself in the chest. It is very rare to meet with tubercles in the intestines of individuals whose lungs contain none of them. Of all the parts of the digestive tube, the end of the jejunum and the ileum are those in which tubercles most frequently develop themselves. We have much less frequently met with them in the commencement of the first of these intestines, as well as in the duodenum. More rarely still are they observed in the cæcum, and in the ascending and transverse colon. We have never found them in the other portions of the large intestine: we once only met with an isolated tubercle in the stomach.

Tubercles constantly arise in the midst of the cellular layers which unite together the different coats of the intestine. Rarely existing in the sub-peritoneal cellular tissue, they show themselves most commonly in the midst of the meshes *(mailies)* of the sub-mucous cellular tissue. At their origin, they appear under the form of white points, having scarcely the volume of the head of a small pin. They are perceptible through the mucous membrane, which above them has preserved its healthy state. They become gradually enlarged, and finally acquire the size of a pea. We have never seen them of a larger size. Under such circumstances, they appear under the form of small round masses, of a dull white, generally isolated from each other, and very rarely agglomerated, like pulmonary tubercles. They project beneath the mucous membrane, which they raise up. The internal surface of the intestine around them is most commonly pale or somewhat injected.

By gently passing above them the blade of a scalpel, the mucous co which covers them may be elevated, issue is given to the tuberculous matter; and in the place which it occupied, a small cavity exists, with white, elevated, and round edges, perfectly similar to an ulceration. Sometimes, in the whole extent of the canal, no other lesions are discoverable than those tubercles, such as we have just described them; they are then in their state of crudity. But in the majority of instances, there exist, at the same time, ulcerations of various forms and sizes, at the base of which the *debris* of tuberculous matter is often discovered. A perfect analogy seems to us to exist between the formation of these ulcerations and that of tuberculous ulcerations of the lungs. In the midst of the parenchyma of the lungs, as well as the intestinal mucous coat, tubercles at first develope themselves, without the tissue surrounding them seeming in any manner inflamed. In the lung, as in the intestine, we frequently see these tubercles exist in the part of this organ which is not inflamed; whilst the parts where we do not meet with them present evident signs of recent or chronic inflammation. To this it may be objected, that where the tubercles are developed a previous inflammation had existed, which, having disappeared in time, had left no other trace than the presence of the tuberculous matter. But this can only be a supposition.

In the lung, as in the intestinal canal, tubercles, in proportion as they become soft, occasion either the erosion and complete destruction of the parietes of the bronchiæ with which they are in contact, or of the digestive mucous coat below which they have taken birth, and eat a route outwardly. At such times, in the parenchyma of the lung, as at the internal surface of the intestine. a cavity exists, the parietes of which become inflamed, and secrete a purulent matter, the quality and quantity of which are very variable. We have frequently witnessed in intestines full of tubercles, ulcerations which had a very great resemblance to pulmonary vomicæ; like the latter, they represented anfractuous cavities, separated by bands of an irregular form.

In some cases, at the same time that the tubercles raise up the mucous membrane which they tend to destroy, they develop themselves also on the side of the muscular coat; separate its fibres, and come in contact with the peritoneum, which, in the same manner as the mucous coat, ends in becoming destroyed. Hence results a solution of continuity of the intestinal parietes, which remains sometimes filled up for a greater or less time by a tuberculous mass. We have observed a similar disposition.

We have just seen that tubercles may arise and develop themselves without previous inflammation, but we frequently also observe traces of an intense inflammation in the portion of intestine where they exist. It is not rare, for example, to find the base and edges of intestinal ulcerations, in individuals labouring under chronic diarrhœa, sprinkled with tubercles still crude. It even seems that inflammation favours their development; and it is almost solely in this case that they are found pressed and agglomerated together.

If we now consider intestinal tubercles as regards the symptoms which announce them, we shall again find several points of relation between them and pulmonary tubercles. Like the latter, they may, when not numerous, exist a long time without any symptom occurring to lead us to suspect their existence. Thus we have found the intestinal mucous membrane raised up by tubercles still small, and a little numerous, in phthisical subjects who have never had diarrhœa: other individuals, whose intestines are in a similar state, have not constantly diarrhœa, but in them it manifests itself under the influence of the slightest cause; exposure to cold or humidity, and the least error in regimen, produce it.

It is thus that the pulmonary mucous membrane is very easily irritated in persons whose lungs contain a small quantity of tubercles still crude. But these multiplied irritations hasten, in their turn, the discharge of the tuberculous matter either into the lungs or intestines. The diarrhœa does not appear to become considerable and permanent until ulcerations have succeeded to the softening of the tubercles. The formation and development of intestinal tubercles are generally accompanied with very little pain: it is the same also with the ulcerations which succeed them. Many patients affirm that they have never experienced any abdominal pain; others feel from time to time slight and very supportable colics. Some, however, do complain of tolerably severe pain. After death, no lesion is discoverable capable of explaining these differences.

We have sometimes observed, in different portions of the small intestines, miliary granulations similar to those which develop themselves in the parenchyma of the lungs. On passing the finger over the internal surface of the intestines, we find a crowd of small rough bodies of the size of a millet seed. Their transparence, their, as it were, cartilaginous hardness, distinguish them from tubercles properly so called, which are soft and opaque. Moreover, they are developed in the tissue of the mucous coat, and not below it like the tubercles, as it is easy to verify by detaching that membrane: they come away with it.

*Scirrhous and Encephalated Tissue.*—We have just seen that the stomach and rectum are the two portions of the digestive tube in which tubercles are most rarely developed. They are, on the contrary, these, where the scirrhous and cerebriform, or encephaloid tissue, most commonly appears.

These tissues may arise primitively, either in the mucous coat itself, or beneath it.

When they arise beneath the mucous coat, we observe them, at first, invading the laminated tissue: they may acquire a considerable size, and form even tumours projecting through the parietes of the abdomen, before being propagated either to the muscular coat, the muscular fibres of which may be easily dissected from beneath them, or to the mucous coat, which is found every where white and sound. But, most commonly, if the cancerous tumour be at all considerable, we no longer observe any trace of muscular fibre; the mucous coat ends in becoming disorganized and destroyed, and the internal surface of the stomach then presents an ulcer of a greater or less extent, the edges of which are formed by the mucous membrane, irregularly divided, *(decoupée)* and the base by the scirrhous and encephaloid tissues, in a state of crudity or softening. In proportion as they pass from this state, they become destroyed, and perforation of the parietes of the stomach takes place sooner or later. But sometimes, as we have already seen, the contents of this viscus are effused into the peritoneal cavity, whence fatal symptoms arise; sometimes, by a happy artifice of nature, cellular adhesions established between the circumference of the ulcer and a neighbouring organ, prevent effusion from taking place. We have frequently seen the liver and the pancreas form the plug. In the cases of this kind which we have observed, we have never found the pancreas diseased. As for the liver, we have sometimes found its tissue uninjured beneath the greyish or reddish *detritus* covering the base of the ulcer; occasionally we have seen it participating in the cancerous degeneration to the extent of some lines only, beyond which it was healthy. Finally, at other times the disease seemed to have been propagated from the liver to the stomach, which latter organ seemed only to have been secondarily affected, and the parietes of which had been destroyed from without to within.

In a small number of individuals, the cancer primitively invades the mucous coat, which becomes thick, soft, fungous, and at last ulcerated. In this manner cancer of the womb frequently commences by ulceration of the mucous coat, lining the interior of the vagina, and the neck of the uterus. No tumour in such case precedes the ulceration. At other times, the cancer, primarily developed in the mucous coat, shows itself under the aspect of a polypiform tumour, projecting above the rest of the membrane, sometimes to the extent of some lines only; at other times, of from one to three inches, connected with it by a large base, or by a small peduncle. Many of these tumours exactly resemble certain champignons, the long stalks of which support a large capital with round edges. Their surface is commonly rugous and unequal, frequently bleeding; their tissue soft, fungous and very vascular. The mucous membrane around them is most commonly healthy. Similar tumours are found in every part of the stomach; we have seen them implanted round the cardia, oppose the free introduction of nourishment into the stomach, and we have observed them around the edges of the pylorus, obliterating more or less completely that opening. When inconsiderable, and occupying the surface of the stomach, they may exist without in any manner disturbing the functions of that organ. We have found them, after death, in individuals whose ready digestion, and the complete absence of pain at the epigastrium, nausea, eructations, &c. did not admit of even a suspicion of the existence of organic disease of the stomach.

*Erectile Tissue.*—We have sometimes witnessed, loose *(flottant,)* at the internal surface of different portions of the intestinal canal, small round or oblong tumours, of a brownish colour, attached to the mucous coat by a thin and narrow pedicle, and having, upon an average, the size of a hazel-nut. On cutting and pressing them between the fingers, some black liquid blood is made to issue; and on subsequently washing them, their tissue is observed to be formed of a crowd of filaments, intersecting each other in different directions, and leaving between them interstices of various forms and sizes, where the blood seems to have been effused. The pedicle of these tumours is formed by a prolongation of the mucous coat, which around them is healthy.

These tumours, which present all the characters of the *erectile tissues*, are but rarely met with: and when they do exist, only one or two can be discovered in the whole extent of the canal. Once we have seen the mucous coat of the cæcum present eight or ten crowded together.

In the cases which we have observed, the *erectile tumours* were too small for any symptom to announce their existence. When more considerable, might they not be the occasion of extensive hæmorrhages?

*Mélanoses*—Under this name we designate small black tumours which we have several times observed in the great intestine. They are subjacent to the mucous coat, which they raise up; those we have dissected were, upon an average, of the size of a hazel-nut. They were round, of a beautiful deep black, crushed easily under the finger, and were like the matter with which common anatomical injections are made. We have found the transverse colon filled with similar tumours in an individual who for a long time had been subject to an abundant diarrhœa, which alternately appeared and disappeared; the mucous coat was in no wise diseased.

The mucous tunic of the small intestine, and of the cæcum, is sometimes found covered by a truly infinite number of small black points, of scarcely the size of the head of a very small pin, and which may be somewhat justly compared to the hairs of a beard recently shaved. We find them, most commonly, crowded together, so as to form by their assemblage round or oval patches, on which the white mucous coat appears as if penetrated by an infinite number of these small spots; their colour may be made to disappear on slight friction: the mucous coat beneath them presents a small lacuna, with a white edge and base.

These black spots sometimes cover the mucous coat to the extent of several feet. It is in the lower quarter of the small intestine that they are most frequently observed. We have never found them in the stomach, and they are very uncommon in the great intestine below the cæcum. They exist in all possible conditions of the mucous coat. We met with them so often during the summer of 1821, in individuals who had died of different diseases, that we are much inclined to think that no morbid symptom is induced by them. Our friend and colleague, M. Descieux, has also observed them in a very great proportion of the sheep killed for the market, the intestinal canals of which he had occasion to examine, towards the end of the autumn of 1821. But what is a remarkable circumstance, we found them but rarely after the commencement of winter!

If we endeavour now to determine the nature of these black points, we may remark, first of all, that they have a disposition similar to that which the mucous follicles present, as so well described by Leger nearly two centuries ago. Like these follicles, they are observed grouped around each other, and forming, by their agglomeration, circular, oval, oblong, angular, &c. figures. Like them, they are found more numerous the nearer we proceed in our examination to the cæcum. Is it not allowable to imagine, from the preceding considerations, that these black points are nothing more than the result of a morbid secretion of the follicles?

*Serous Cysts.*—We have frequently found these cysts developed beneath the mucous coat, both in the small and large intestines. The major part of those which we have met with were of small size. The largest we have had occasion to dissect was of the size of a common walnut. Primitively developed in the laminated tissue, subjacent to the mucous coat, it had extended between the fibres of the muscular coat, and formed a remarkable projection beneath the peritoneal coat.

If we are to believe authors, this species of cysts have, under some circumstances, undergone a prodigious development. Bonet, Dehaen, and Peter Frank, have related cases in which these tumours had been mistaken for asciatic dropsies; the operation of paracentesis was performed: an enormous quantity of serosity issued from the abdomen, and it was not until after death that the true nature of the disease was discovered.

Several authors have likewise spoken of vesicles full of a serous liquid, developed at the free surface of the mucous coat, to which they were adherent. Bonet observed one of these vesicles, seated on the pyloric circle, and projecting to the extent of half a finger's breadth into the cavity of the duodenum. The mucous coat of the stomach, in its whole extent, presented several others of them, some of which were whole, and others lacerated. Bonetus gives them the name of hydatids.

*Œdema of the Intestines.*—Not only do serous cysts develop themselves at the free or adherent surface of the mucous coat of the intestines, but also the laminated tissue subjacent to that membrane may become the seat of a real serous infiltration. We have several times observed this œdema of the sub-mucous cellular tissue, the existence of which had been denied by Bichat. We have met with it more especially in dropsical subjects, who had laboured under diarrhœa, in aged people, who, after having languished for a considerable period in the hospital, had finally fallen into an adynamic state: and in patients exhausted by chronic organic affections. We observed it particularly well marked in a female who had laboured under encysted dropsy of the ovary.

We have also witnessed a considerable quantity of serosity effused between the laminæ of the cellular tissue subjacent to the mucous coat, lining the internal surface of the gall-bladder.

*Adipose Tissue.*—We have only once met with a fatty tumour in the substance of the parietes of the digestive tube. It existed towards the middle part of the ileum, and was of the size of a hazel-nut. Developed beneath the mucous coat, which had preserved its natural appearance, it presented a uniformly smooth surface; but when the mucous coat covering it was removed, it presented a clustered appearance *(un aspect comme pelotonné.)*

It was perceptibly formed of a numerous assemblage of small oval or spheroidal vesicles, separated by slight furrows, traversed by vessels; each of these vesicles contained fat. A narrow pedicle kept the whole tumour adhering to the laminated tissue.

Section III. *On the state of the digestive tube in the different fluxes, known under the name of diarrhœa, dysentery, and lientery.*—These affections have been for a long time looked upon as diseases entirely independent of inflammation of the intestines. Many ancient authors have, in truth, spoken of the ulcerations seated at the internal surface of the digestive tube in chronic diarrhœas, but they consider them as an effect of the diarrhœa. Such was the opinion of Boerhaave, and of Van Swieten, his commentator. We have already seen that such also was pretty nearly the idea of Stoll; and it may be likewise found in the writings of Hippocrates. He was not ignorant that in dysentery the intestines are the seat of more or less deep ulcerations, but he regarded them as being occasioned by degenerated bile and phlegm.

Is relaxation of the bowels constantly connected with an inflammatory condition of the mucous coat of the intestines? This is a very important question in a therapeutical point of view. We shall endeavour to answer it by presenting a summary of the numerous cases which we have collected on the subject.

We have several times found, in individuals who had laboured under recent or chronic diarrhœa, the internal surface of the intestinal canal very pale in its whole extent, the mucous coat having preserved its thickness and ordinary consistence. Patients weakened by long organic diseases, hydropics, old people under that state of debility denominated by the ancients cachexia, and who succumbed after having laboured, for a longer or shorter time, under considerable looseness, frequently present that state of the intestinal canal. Their stools are copious, very liquid, purely watery; surpassing by much the quantity of iquids taken in. We have sometimes found, in cases of this kind, a well-marked serous infiltration of the sub-mucous cellular tissue.

Morgagni has transmitted to us the history of several cases of diarrhœa without inflammation of the mucous coat of the intestine. He has seen several individuals labouring under that disease die in a short space of time, exhausted by the excessive abundance of their alvine evacuations. In these atonic diarrhœas, the intestinal parietes frequently become much extenuated; the fleshy coat, especially, experiences a true atrophy, and occasionally seems to be only composed of some pale and small fibres, widely separated from each other. Bonet had already remarked this fact. "In chronic diarrhœa," says he, "we find the intestines as thin as a cobweb." The intestine, in this state, seems to be unable to fulfil its functions; chylification is but imperfectly performed, absorption becomes much less active, and the food is frequently voided as when taken. This is what the ancients designated under the name of lientery.

The mucous coat of the intestines may then, like many other textures, become the seat of a much more abundant secretion than usual, although it presents no trace of inflammation. It is thus that, during convalescences from chronic maladies, the exhalation of serosity into the subcutaneous cellular tissue is frequently augmented. It was not, therefore, without reason that Sauvages designated under the name of *flux*, a particular class of diseases.

Since truly atonic fluxes may exist, it follows that a strengthening and astringent treatment is, in these circumstances, the only proper plan to be pursued. Thus the œdemata, of which we shall presently speak, may be dispersed, either by the employment of topical stimulants, or by the internal administration of tonic medicines.

In other individuals, we find the mucous coat of the intestines equally white in its whole extent; but beneath it numerous tubercles, or other adventitious tissues, exist. They provoke diarrhœa, either by the sympathetic irritation which they determine to the mucous membrane covering them, or by stimulating by their presence the muscular coat, the contractions of which consequently become more rapid and more intense. It is in this manner that the different adventitious tissues, developed in the parenchyma of the lungs, provoke an habitual irritation of the mucous membrane of the bronchiæ; but, most commonly, the diarrhœa in this case does not appear to become permanent and considerable, until the period when the tubercles, having become softened, inflame and ulcerate the mucous coat.

It is, moreover, indubitable, that in a very large majority of cases, the intestines of individuals labouring under diarrhœa, whether complicated or not with dysenteric symptoms, present evident marks of phlegmasiæ.

This phlegmasia may be seated in the small or in the large intestine.

In the small intestine, it frequently only exists to the extent of some fingers' breadth above the ileo-cæcal valve; at other times a larger portion of the small intestine has laboured under it, where it either appears under the form of a simple injection of the mucous coat, alteration of its texture, red or white softening of its tissue, or ulceration.

Numerous cases have apprised us that acute or chronic diarrhœa is the frequent result of isolated inflammation of the small intestine, without the large participating in it in any manner. We insist on this fact, because M. Broussais has laid down, as a general principle, that enteritis is accompanied with constipation, and that diarrhœa only supervenes when enteritis is complicated with inflammation of the colon, *(colite.)*

Of the three portions of the great intestine, the cæcum is that which, in diarrhœa, most frequently presents one of the three degrees of inflammation; after that the colon; and, lastly, the rectum.

The symptoms, the *ensemble* of which constitutes dysentery, are not connected with any particular state of the intestines. The tenesmus alone announces that inflammation exists in the rectum. As for the bloody and glairy stools, they appear in individuals whose intestines present lesions analogous to those which are observed in other patients whose stools had always been purely watery.

We once found a somewhat considerable number of ulcerations in the ascending colon, in a phthisical patient, who, after having previously laboured under diarrhœa, had not felt it for a long period, and had even become constantly constipated. It may be conceived that this may be the case, when the ulcerations are small, by no means numerous, and when neither the edge nor the base is inflamed. In fact, at such times they can only, like tubercles, produce a flux by the sympathetic irritation of the mucous coat surrounding them, or of the muscular coat.

The different states which the digestive tube may present in diarrhœa being well known, can they be distinguished during life from the symptoms which manifest themselves? This, in many cases, is possible. Thus, if pains of the abdomen are observed, if the skin is burning hot, the pulse frequent, if the dejections are slimy, membraniform, or bloody, we may be satisfied that the intestine is the seat of more or less intense inflammation.

We may add, however, that nothing is more common than the absence of every species of pain in cases where numerous ulcerations cover the internal surface of the intestines, whether of the ileum, cæcum, or colon. How frequent, also, is it, on the other hand, to see patients complain of violent pains in the abdomen, although the digestive mucous coat is not the least inflamed. Is not this the case, as the success of the treatment confirms, in lead-colic, which is cured by the employment of the most active drastics, in the colics termed nervous, which frequently yield to eminently stimulant medicines, and in those which are owing to accumulation of flatus and of fæcal matters, and which are treated with so much advantage by repeated purgatives? Stoll has cited a remarkable case of syphilitic intermittent colic, which yielded to the use of corrosive sublimate.

We have already seen that intestinal tubercles may arise, become developed and softened, without any pain indicating their presence.

The character of the stools is not always itself a certain sign by which we can recognize inflammation. Sanguineous evacuations have been observed to take place *per anum* in individuals whose intestinal mucous coat was found sound after death. These passive hæmorrhages are analogous to those which take place in many dropsical individuals, at the internal surface of the serous membranes of the chest and abdomen; they are similar to the hæmorrhages of which the skin, the cellular tissue, and the synovial membranes, become the seat in scorbutics.

The serous dejections, resembling water, coloured yellow or green, manifest themselves equally in all possible conditions of the digestive tube, in the cases where it is ulcerated, and in those in which its parietes are pale, thin, and œdematous *(infiltrées.)*

When even ulcerations exist in the intestines, ought they to be regarded as a constant obstacle to the employment of tonic and astringent substances? They present such a great variety in their nature, that it seems the same method of treatment cannot agree with all.

The white, grey, or brown colour of the base, the nature of the secretion which takes place there, the want of, or the considerable thickening of the laminated tissue which forms it, the appearance and disposition of their edges, the different degrees of consistence, of thickness, and of colour, of the mucous coat which constitutes them, the separation of this membrane to a greater or less extent, its state in the spaces between the ulcerations—are they not so many circumstances which seem to demand a multiplicity of modifications in the treatment? We may thus easily explain how any curative method succeeds very well in one case, and completely fails in another. We have seen, for example, several diarrhœas yield to the decoction of catechu; we have seen others increase, and become aggravated during the administration of this medicine, although in both cases the symptoms were nearly the same, and the patients placed in nearly similar general circumstances; the major part were consumptive individuals. It would frequently be of great importance, could we, in the same portion of intestine, apply an astringent or tonic substance upon the ulcerations, and cover with emollient applications the spaces which separate them, and reciprocally. In this manner the Surgeon acts in the treatment of several ulcers situated on the surface of the body. He heals them by endeavouring to keep up inflammation to a certain degree, above and below which the disease cannot proceed towards resolution. Is it not again by the employment of topical astringents that many chronic ophthalmiæ are cured? Is it not also by the employment of resinous substances that chronic phlegmasiæ of the mucous membrane of the lungs and urethra may be very successfully treated? We have very frequently seen M. Lerminier have recourse, with marked advantage, to a slightly stimulant treatment towards the end of acute pneumonia, having a tendency to pass into the chronic state.

Finally; in order to add fresh weight to these considerations, we might invoke the authority of the ancients, who, in diarrhœas and chronic dysenteries, made a frequent and successful use of many astringent substances, given under different forms.

We shall not suffer ourselves, however, to accumulate observations for the purpose of elucidating these important questions, but conclude with the following quotation from Mushenbroek:—

"*Pauca experimenta nos confidentes reddunt, audaces, gloriosos; multa incertos; plurima denique, ac humanam ferè superantia patientiam ad aliquid concludendum nos eminùs præparant.*"*

---

2. *Leeches.*† Leeches should not be much handled prior to their application. If the weather be cold, they are liable to become torpid, and should be warmed by breathing on them.

Leeches may be applied by means of a wine or cupping glass. It is useful, in either case, to put a piece of stiff paper to the bottom of the glass, to which they are otherwise apt to retire; or, if they are required on a particular spot, a small glass tube, four or five inches long, and just large enough to admit the body of the leech, may be employed. If the leech remains torpid during the process of sanguisuction, a drop or two of cold water sprinkled on it will arouse it again into action. The best methods of suppressing hæmorrhage, which sometimes succeeds to their application, and is occasionally very troublesome, when all the simple means, such as felt, starch, puff-ball, &c.

---

* The following is an extract from the "*Report made to the Section of Medicine of the Royal Academy of Medicine,*" *by M.M. Chaussier and Hippolyte Cloquet, on the preceding Mémoire;*—"En somme, Messieurs, nous avons trouvé dans le Mémoire qui nous a été soumis, les preuves d'un grand éloignement pour tout esprit de système; il est propre à donner de son auteur une très favorable idée, par le soin assez scrupuleux que méritoire, avec lequel celui-ci a fait ses observations; la forme d'ailleurs répond ici au fond, et nous concluons à ce que le travail dont nous venons de vous rendre compte soit honorablement déposé dans vos archives, jusqu'à ce que l'Académie s'occupera de compléter le nombre de ses membres, le nom de M. Andral, fils, devra être porté sur la liste des candidats.

" Paris, le 30 Juillet, 1822.

" Signé CHAUSSIER.
HIPP. CLOQUET, Rapporteur.

" Certifié conforme par le Secrétaire de l'Academie,
" BECLARC."

† Dr. Rees Price.

have failed, are to apply the spirits of turpentine, or the muriated tincture of iron; or, in failure of these, to insert a piece of lunar caustic, (the point being scraped very fine,) into the puncture; or, lastly, a weak solution of tartarized antimony has lately been recommended as very efficacious. Dr. Price says that, in order to preserve the leeches, they should be suffered to retain the blood, and merely be thrown into a jar of fresh water. In the course of a month or two, they will become firm and healthy, and able to perform their functions again; but, if the time cannot be spared for their recovery, he prefers what is called *stripping them*, to making them disgorge the blood by means of salt. It seems that if the blood is carefully squeezed from the leech, it is capable of resuming its functions immediately.

---

3. *Meningine.* The farther we see into the animal economy the more we shall be convinced of the intimate sympathy (or whatever name the connexion may receive) between the alimentary tube and the sensorium. Dr. Scoulton, of Metz, has published in the Journal Universel des Sciences Medicales some anatomico-pathological researches on this subject, for the purpose of illustrating the connexion between irritation of the mucous membrane of the intestines and the pia mater. He thinks that diseased states of this last-mentioned delicate membrane have been often overlooked, in consequence of the changes in its struc ture not being very conspicuous to the hasty and superficial observer. We conceive, however, that the changes of structure in this tissue are more easily detected than those in most other parts of the body. In a state of health this web is perfectly transparent, thin, and colourless, without adhesions. Any deviation from this state must therefore be one of disease. It is no doubt desirable, but seldom possible, to have a healthy brain to contrast with an unsound one when we are prosecuting post-mortem researches. This was the plan pursued by Wenzel in examining the brains of epileptic patients. Dr. S. thinks that all parts of the intestinal canal do not sympathise equally with the brain. The stomach and small intestines possess a much closer affinity with the meninges than the great intestines. So it is with respect to the different coats of the intestines. While inflammation of the mucous membrane gives rise to corresponding disturbance in the pia mater, this last remains free from all sympathy in peritonitis. When the mucous membrane of the stomach or small intestines is inflamed, Dr. S. has observed the vessels of the pia mater injected with blood, forming red patches, and sometimes amounting to extravasation. These appearances are chiefly found in the anterior and lateral parts of the brain —unless the abdominal inflammation be very violent, in which case nearly the whole of the pia mater is affected—its minute vessels carrying red blood, and giving rise to effusion and adhesions. Dr. S. thinks that these views are likely to bear on and elucidate many of the phenomena of apoplexy, on the principle of every inordinate excitement of the stomach being communicated to, and, as it were, repeated in, the brain, thus occasioning determinations of blood to the head, and rendering its vessels gradually more debilitated and ready to give way under any particular increase of action.

---

4. *Poisoned Wound.** Mr. Wansbrough, of Fulham, assisted in examining a body in the month of June, 1822, the corpse being very

* Mr. Wansbrough. Med. Repos. No. 113.

obnoxious. On the same day he pricked his finger with a thorn, which was followed by great pain, inflammation, and swelling, not only of the finger, but of the hand and arm as far as the elbow. The distressing symptoms continued nine days, during which there was excessive derangement of the nervous system, the powers of the stomach being almost entirely suspended, and the tongue covered with a thick white coat. All medicine was rejected, except calomel, which acted on the bowels, and kept them in an open state. He slept but an hour each night, from twelve till one. There was scarcely any fever during Mr. W's illness—but there was a copious flow of saliva from the mouth, not the effect of the calomel. In three weeks he was able to seek change of air, and a residence of a few weeks at the sea side completed his recovery. The attack was succeeded by a fall of the hair from the head. From all the circumstances we have some doubt if the accident was owing to the absorption of morbid poison at the time of the prick from the thorn. It is impossible, however, to be certain on this point. We congratulate Mr. W. on the improved state of health which succeeded this shock, and which has enabled him to conclude his paper with the consolatory reflection that—" out of evil proceedeth good."

---

5. *Abortion.** Notwithstanding the redundancy of our population, it is still the duty of the accoucheur to bring as many living children into the world as possible; and consequently the means of lessening the frequency of abortion are very desirable acquisitions. It appears to have been the opinion of the late Sir Richard Croft that diarrhœa generally precedes or accompanies abortion. Dr. D. Stewart, an able accoucheur and lecturer, seems to think irritation in the bowels a very common *cause* of the accident. To this latter gentleman Mr. Ward admits his obligation for a knowledge of the efficacy of opiate suppositories in preventing the death of the fœtus in utero. Some time ago Mr. Ward was called to attend a lady who was in the fifth month of pregnancy, but without any pains warranting the idea of labour. He was informed, the patient had never borne a living child, but had aborted three successive times, at the period of gestation abovementioned. The present symptoms resembled those experienced on former occasions. A diarrhœa had existed for four days. An aromatic cretaceous mixture was ordered, with opium; but in less than eight hours a dead fœtus was expelled. " The placenta was retained, as is common in such cases, longer than is usually the case in natural labours, and after waiting an hour, he extracted it."† No untoward symptoms occurred, and the patient recovered.

Our author was again called to the same patient in the fifth month of pregnancy, and labouring under precisely the same symptoms as before detailed. A laxative draught with castor oil was ordered, which produced no alteration in the nature or quantity of the alvine evacuations, and therefore the cretaceous mixture with opium was ordered. Still the symptoms continued. The cretaceous mixture was now laid aside, and opiate suppositories were had recourse to, but without the

---

* Mr. H. W. Ward. Med. and Phys. Journal, No. 289.

† If the placenta is detained in utero, in cases of abortion within the sixth month, we really cannot easily conceive how accoucheurs can introduce their hands so readily as they say to extract the secundines. If the placenta be merely in vagina, it is another affair.

wished for effect. Large and repeated doses of opium were therefore exhibited by the mouth, with the view of allaying irritation. This measure had the desired effect of subduing the bowel complaint, and the patient went through the usual term of utero-gestation, with final delivery of a living child.

Viewing intestinal irritation as an occasional cause of abortion, we cannot object to the measure here enforced by Mr. Ward.

---

6. *Amenorrhœa.* Dr. Lavagna has recently published, in a Milan Medical Journal, an account of his success in provoking the menstrual discharge by means of injections into the vagina, composed of ten or twelve drops of the volatile alkali to two table spoonfuls of warm milk. This injection he uses three or four times a day, and in every case it brought on the catamenial discharge in the course of five or six days after the commencement of the remedy. The injection produces generally a disagreeable sensation in the vagina, but in no instance was it productive of any bad consequences. Fourteen cases are related in illustration.—*Rev. Med.*

---

7. *Epilepsy.** A soldier who had become paralytic of the left side while serving in the Peninsular war, was seized with epilepsy on Christmas eve, 1820, for the first time. At first the fits were very frequent; but by tonics combined with occasional aperients and local bleedings, they were rendered less frequent. Pills of croton oil acted very powerfully on his bowels, and the paroxysms were reduced to one in two or three weeks. But the croton oil lost its power, and the man became affected with pectoral complaint in addition to his other ailments. At this time, (31st October,) Dr. Carter ordered the tartar emetic ointment to his chest, which produced sloughing of the integuments and a profuse discharge. The eruption also spread over the whole of the trunk, and caused excessive irritation for several days—they died away. The epileptic attacks did not return; but in about two months he had some premonitions, which induced Dr. Carter to order leeches to the temples, and the application of the ointment to the left arm, as he would not consent to have it again on the chest. It produced great irritation and swelling in the whole arm, with profuse discharge. At the end of five months, when the report closed, there was no return of epilepsy. We have no doubt that this suspension may be fairly attributed to the antimonial irritation and consequent discharge. We are glad to learn that Dr. Carter is prosecuting his enquiries respecting the utility of antimoniated ointment in epileptic affections.

---

8. *Tic Douloureux.*† We are rejoiced to see that accounts still continue favourable to the reputation of carbonate of iron in this dreadful disease. In a late number of our respected cotemporary, Dr. Crawford, of Bath, has communicated the particulars of two cases of neuralgia treated with the remedy in question—one in his own practice, the other in that of Dr. Davis of Bath. The first case was that of a lady

---

* Dr. Carter. Med. Repos. No. 113.

† Med. and Phys. Journal, February 1823.

who became affected in the left side of her face at the age of 69 years. The disease was moderate at first, and the intervals between the paroxysms of considerable extent; but after some time the disease became greatly exasperated, and the cessations from suffering of short duration. The complaint was subdued by the arsenical solution, and the patient remained free from sufferings for two years. Then a slight relapse, and again relief for four years by the same remedy. At the end of this period the enemy renewed his attacks, but arsenic no longer availed. Dr. Crawford then commenced with carbonate of iron in scruple doses three times a day, gradually increasing the dose to a drachm. In three weeks from the commencement of this plan of treatment the disease gave way, and has not yet returned.

Dr. Davis's patient was also a female, 65 years of age, affected with the diseases in the right side of the face. She was cured by the carbonate of iron in a fortnight—two scruples night and morning.

Mr. Thomson, of Sloane Street, has communicated to Mr. Hutchinson the particulars of some cases which are in favour of the remedy. We sincerely hope it may retain the reputation it has already acquired.

---

9. *Antimoniated Ointment in Agues.** During the winter of 1815, Dr. Pommer had a great number of intermittents under his care, while the army of Wertenburgers was quartered on the Loire and Allier. As the bark often failed, he was led to try the effect of bringing out crops of pustules on the abdomen with the tartarized antimonial ointment; and, to his surprize, in every instance the fever, whether tertian, quartan, or irregular intermittent, gave way soon after the pustules were completely formed.

We have, of late years, found this ointment so useful in many anomalous affections, that we consider it a very important item in the Methodus Medendi. The following is the form which we have found most useful and quick in operation;—

R.—Axungiæ, ℥j.

Antimon. Tart. ʒiij.

Tinct. Lyttæ, gt. xx.

Misce fiat unguentum. A sixth part of the above is generally sufficient to be rubbed on any part every night, and in three or four frictions a copious crop of pustules will appear. In several instances it has brought out many pimples on the scrotum when rubbed on distant parts of the body.

---

10. *Destruction of a Portion of the Spinal Marrow.*† This case is so remarkable that it will be entitled to a place among the "*cas rares*" of the next Dict. des Sciences Medicales.

A gentleman, 44 years of age, was the subject. He had led a very debauched life, and at the age of 34 first began to experience pain in moving his arm, attended with uneasy sensations at the top of the spinal column. These symptoms increased, and in January 1815, the use of the member was lost. The dorsal part of the spine now became curved, and the shoulders, particularly the right, raised. Cauteries, moxas,

* Graaffe and Walker's Journal, 1823.

† M. Rullier, and M. Magendie, Journal de Physiologie.

blisters, without benefit. M. Rullier was consulted on the 5th October, 1822, and found the patient emaciated and weak, the size of the head contrasting with the wasted trunk—the spine curved—no loss of voluntary motion, except in the upper extremities. The patient could walk about, even till nearly the period of his death. The arms were permanently and rather painfully contracted—the forearm being in a state of involuntary pronation, the fingers fixed, but more firmly during sleep. Notwithstanding this state, the patient could still write his name by a movement of the whole arm. The hands were morbidly sensible to the *slightest* external impressions, and the arms painfully so to *forcible* impressions *ab externo*. The intellect was unaffected; but his sleep was disturbed by uneasy respiration, palpitations, and painful stitches in the chest. There was cough and cream-like expectoration—hectic fever—bowels constipated—violent pains in the lumbar region; the breathing very much embarrassed during the febrile paroxysm. The venereal appetite was unimpaired till the last—indeed it appeared to be unnaturally excited. On the 31st October, 26 days after M. Rullier first saw the patient, death put a period to his sufferings.

*Dissection.* The body was opened 36 hours after death, in the presence of Magendie, Piedagnel, and Leconteux. The chest and upper extremities were in a state of the most complete marasmus—the legs and feet œdematous. The upper part of the dorsal spine presented a slight salient curvature backwards and to the right, which raised the corresponding shoulder. The rest of the back appeared natural. The brain was sound, but contained much water in the ventricles, which seemed to drain into the spinal arachnoid cavity when the body was raised erect. There was nothing about the cerebellum which indicated the salacity of the patient during life. The cavity of the spine was laid open throughout its whole extent, by removing the spinous processes and plates of the vertebræ. The medulla spinalis suffered no compression, but only contorted itself in conformity with the spinal column in the dorsal region. The arachnoid cavity contained much serosity. The spinal marrow, examined from behind, appeared in its natural state, as far as the 4th pair of cervical nerves. The two inferior thirds of its dorsal portion also seemed sound; but a remarkable alteration presented itself between these two parts, and corresponding to the eighth or ninth pair of nerves. It was soft even to fluidity, so that it flowed about, according to the position of the body. The coverings being slit open over this portion, a quantity of fluid ran off, mixed with flakes of medullary matter. The parts then presented to their view an elongated cavity, filled with a greyish red fluid, in which were dispersed a great number of extremely thin capillary vessels. The medullary bands connected with the corresponding roots of the spinal nerves could scarcely be distinguished in the anterior part of the altered portion. On the anterior face of the spinal marrow, this disorganization was not near so great. The medullary bands corresponding to the reticulation of the origin of the anterior branches of the spinal nerves were apparent, and offered no interruption throughout their whole length, with the exception of the left, which was altered.* They were traced throughout the whole extent of the marrow as far as the medullary tissue, whence they derive their origin. The portions of medulla spinalis above and below the part now described, shewed nothing worthy of observation.

Dr. Magendie has made many interesting remarks on this curious case. Here, says he, we see a man enjoying almost to the last hour

* The anatomical description is very confused in this part of the original.

great moral energy, powerful generative faculties, a free use of his lower extremities, and *feeling* in the upper ones, yet with the loss of nearly a third of the spinal marrow! The communication between the cerebral and dorsal part of this marrow was apparently only by the investing membranes; for there remained only a thin layer of white substance scarcely two lines in breadth, and that too, very probably, altered in structure. There was, therefore, an almost complete isolation of the superior and inferior parts of the marrow—and that for a space of six or seven inches. Yet the will exercised its influence over the lower extremities—the imagination stimulated the genital organs, and these transmitted to the sensorium the lively emotions of pleasure. The great sympathetic nerve was not, he thinks, the medium of communication between the upper and lower portions of medulla spinalis; for all sections or even compressions of the marrow intercept the determination of the will relatively to the motions, and render the parts which receive their nerves below the point compressed insensible. The thin layer of medullary matter then, and the membranes only remained. The contraction of the upper extremities, with a continuance of sensibility, deserve to be remarked; for the posterior portions of the marrow, where the sensibility particularly resides, had disappeared from all the pairs of nerves which supply the brachial plexus. Thus the sinsibility of the arms could not have had its usual source; viz. that which is connected with the posterior roots. In short, M. Magendie seems at his wits' ends to reconcile this piece of pathology with his former physiological experiments—and, as M. Rullier says, "it is evident that we have still much to learn respecting the functions of the spinal marrow."

11. *Extirpation of the Thyroid Gland.** Success does not necessarily warrant an operation—we mean, success in one or two instances. A man recovered after the shaft of a gig had run through his chest, and pinned him to a wall; but not one in 500 would recover under such circumstances. In the case of bronchocele, we do not think that any thing can warrant extirpation:—for if the gland be not very large, such a terrible operation ought not to be thought of; and if it be very large, the hæmorrhage must be dreadful, and leave scarcely a ray of hope for the patient.

It has been stated, however, by Hedenus, of Berlin, that he has operated *successfully* in six cases—one of which he has given in detail. We confess we are somewhat of Wickmann's way of thinking, who says that to attempt to extirpate a Bronchocele, or scrofulous thyroid gland, is nothing else than literally and in good earnest to cut the patient's throat. Hedenus observes that he never was less than half an hour in performing this operation. In the case detailed, he was an hour and a half, and was obliged to tie 63 vessels. After all, there was great hæmorrhage from innumerable vessels which the ligature could not command.

The patient was a young man, 21 years of age, of scrofulous constitution, who had had bronchocele from his youth, and which had now attained the size of a skittle-ball, covering the whole of the fore part of the neck, from the os hyoides to the manubrium sterni, and on both sides pushed back the sterno-cleido-mastoid muscles and parts adjacent. Its circumference at the base was fourteen inches;—its transverse diameter seven inches. It was firm, tense, and heavy; the surface abounding

* Graafe and Walther's Journal. Quarterly Journal of Foreign Medicine, No. XIX.

with varicose veins. It was attached firmly to the larynx, and encroached upon the trachea, exciting a distressing rattling respiration, with threatening of suffocation, especially on quick walking, or ascending stairs. He was operated on in the theatre of the hospital at Berlin, on the 8th October, 1800, in the presenee of numerous spectators.

"The patient was laid on a mattress. I considered the recumbent posture as most convenient to the operator, and comfortable to the patient. I then divided the skin in a longitudinal direction with a convex edged bistoury from the os hyoides to the top of the sternum: not being able by this means to form any flap, I dissected back the skin and platysma myoides on both sides from above downwards, so as to form a flap of two inches, turned back on either side. I then saw the sterno hyoid and sterno thyroid muscles much increased in size, and firmly fixed to the whole tumour, and destitute of the usual aponeuroses: these I endeavoured, for the sake of sparing them in the operation, to dissect and separate from the swelling. Scarcely had I dissected away a quarter of an inch, when blood streamed in great quantity from numerous small arteries, which could neither be tied on account of their minuteness, nor restrained by any styptic application, since their mouths were retracted and hid beneath the strong and firm aponeurosis which invested the tumour, so that they could not be reached or withdrawn. I gave up, therefore, the attempt to separate these muscles, and came immediately to the resolution of cutting them through at their points of attachment above and below, and removing the included portions with the tumour. I next, continues our author, separated the swelling above and below, from the sterno cleido mastoid and omo-hyroid muscles, as also from the jugular veins and carotid arteries (which were intimately connected with the tumour by cellular membranes,) until I freed them as far as the point where the thyroid arteries come off: I then tied both the superior and inferior thyroideal arteries of either side, close to the tumour, and on account of the free anastomoses, used the double ligature, dividing the vessels in the space between. The deeper I carried my dissection now the more serious did the operation appear, since at every cut of four or five lines I was obliged to tie two or three arteries; and these ligatures, cn account of the size of the tumour, and the deep situation of the vessels, were very difficult, and required great care.

"When I had, with the greatest caution, dissected to the base of the tumour, which was firmly fixed to the thyroid cartilage, and the three upper rings of the trachea, on every side so great a number of arteries presented themselves, for the most part of the diameter of the radial or digital arteries, that I could not cut a line or two without dividing two or three of them. Under these circumstances, and as the patient, notwithstanding the speedy ligature of the arteries (effected partly by means of the artery forceps, partly by the needle and thread,) had already lost a very considerable quantity of blood, I hesitated, not that I might bring the operation to a more speedy close, and spare as much as possible the loss of blood, to adopt at once the method since recommended by Brunninghausen, namely, to tie the base of the swelling, and then with the bistoury to extirpate the tumour above it. I used for this purpose a blunt pointed aneurismal needle, armed with two four-threaded ligatures, and while I directed the tumour to be pulled upward, passed the needle through the middle of its base, and drawing the ligatures through, I tied one below, the other above, as firmly as possible. However, that I might perfectly command the bleeding, I passed also a third ligature around the whole circumference of the swelling, and extirpated the tumour now without any bleeding from the parts included in the ligature. I now cleaned the wound from blood with a sponge, that I

might see if any important vessel was yet bleeding ; and as this was not the case, there being only an oozing from the small vessels of the wounded surface, I placed the ligatures over the lips of the wound, and fastened them there by means of plaster. I sprinkled the whole wounded surface with powdered gum arabic, and laid on this agaric wet with Theden's vulnerary water, and filling the remaining cavity of the wound with charpie, I brought the lips of the wound as much as possible together by adhesive plaster ; over the plaster I laid dossils of lint and compresses wet with vinegar, which were renewed every six or eight minutes, the whole was secured by a bandage."

Considerable hæmorrhage ensued, but was ultimately restrained. Great symptomatic fever and other bad symptoms followed, which nearly proved fatal. He was discharged cured (as is said) on the 24th of the succeeding month.

We think, after giving the steps of this operation, few will be inclined to follow the example of Dr. Hedenus in this country.

---

12. *Nervous System.** M. Flourens has made deep researches and many experiments on the properties of the nervous system—or, in other words, on sensibility and irritability. On these researches and experiments, Portal, Pinel, Cuvier, and others, have made a favourable report to the Academy of Sciences. A full translation of the report is given in our respected cotemporary, the Medical and Physical Journal for June of the present year, to which we refer for particulars. We shall here, however, dedicate a page to the conclusions drawn from the experiments and researches.

" According to M. Flourens, there are two properties essentially distinct in the nervous system ; the one to excite muscular contraction, the other to perceive impressions. The object was to determine experimentally what parts of this system serve exclusively for sensation ; and what, on the contrary, serve exclusively for contraction.

" It is evident that the trial of each part could alone ascertain its property. M. Flourens has therefore subjected to trial, separately and in turn, the nerves, the spinal marrow, the medulla oblongata, the tubercula quadrigemina, the cerebellum, and the cerebral lobes. From these experiments, thus chalked out, it follows—

" 1st. That the nerves, the spinal marrow, the medulla oblongata, and the tubercula quadrigemina, are capable of exciting muscular contractions.

" 2d. That the cerebral lobes and the cerebellum are not capable of exciting them. Haller and Zinn had formerly noted the impassibility (insensibility) of the upper layers of the cerebral lobes ; Lorry, that of the corpus callosum ; M. Flourens has, for the first time, observed this insensibility in the whole of these lobes, in the cerebellum ; and has been the first to fix the limit at the tubercula quadrigemina.

" The irritation of a nerve, separated from the nervous centres by section or ligature, is confined to the excitement of abrupt and partial contractions in the muscles to which this nerve is distributed. The nerve, therefore, excites properly only contractions.

" The spinal marrow being cut successively above the posterior enlargement, above the anterior, and near to the occiput : at first the animal lost the use of its hind paws, then of its fore paws, and next of the

---

* M. Flourens. Journal Comp. March 1823. London Med. and Phys. Journal, June, July.

trunk; but, in all these cases, all these parts, the hind and fore paws, as well as the trunk, preserve their collective movements, *(mouvemens d'ensemble.)*

"We ought to add, that these movements take place only in consequence of external irritations. What has disappeared is, first, the co-ordination (consentaneity) of the movements in leaping, flying, walking, standing, catching, &c. and, secondly, the volition of these movements.

"What remain are the contractions, and the connexion of these contractions in associated movements. The spinal marrow, then, properly ties the muscular contractions, in associations *(mouvemens d'ensemble)* as to volition and the co-ordination of these movements that resides elsewhere.

"The irritation of the spinal marrow constantly occasions violent convulsions: its destruction speedily brings on death; but this last effect depends on its action on the involuntary movements. Constantly, the abstraction of one of the tubercula quadrigemina causes the sight of the opposite eye to be lost. The irritation of a tuberculum determines contractions in the opposite iris; its complete removal abolishes the contractions completely. In the tubercula, therefore, the primary principle of the action of the iris and of the retina resides.

"In proportion as we cut off the cerebellum by successive layers, the animal loses gradually the faculty of flying or running; then that of walking; and, finally, that of standing upright.

"A single cerebral lobe being removed, the animal loses immediately the sight of the opposite eye; but the contractility of the iris of this eye continues, notwithstanding the animal experiences at first a weakness much more marked on the opposite side of the body. In other respects, it goes on as usual. The two lobes being removed, there is no longer any vestige of volition, or of memory, or of any perception: memory, volition, perception, reside then in the cerebral lobes."

---

13. *Enlarged Heart.** Pulsations communicated from the heart or large vessels are often very puzzling to the practitioner, and sometimes lead him to erroneous conclusions. Epigastric pulsation is very common in most cases of enlargement of the heart; but a pulsation in the region of the liver is rare, and can only take place from a malposition of the heart, as from an enlargement of the liver in a particular direction, complicated with hypertrophy of the heart. The following case recorded by Mr. Ward, apothecary to the Westminster Dispensary, illustrates these remarks.

A stout man, 33 years of age, had been for two years often affected with dyspnœa, cough, and pain in the chest. These subsided, but left a pain in the right hypochondrium, attended by a troublesome cough and unpleasant sensations in the chest. The breathing became very difficult, especially in the recumbent posture, the pulse being upwards of 100 and wiry. His countenance evinced great anxiety—the lower extremities became œdematous—the urine scanty—and there was a distinct pulsation not only at the scrobiculus cordis but in the right hypochondrium. He died, of course, and on examination the heart was found greatly enlarged, weighing 28 ounces. The lungs were healthy, but the right lobe of the liver "was nearly three times its usual magnitude." When the chest was opened, a large tumour was observed in the lower portion of the right side, "resembling a second heart covered with its pericardium." This, on examination, proved to be the right lobe of the liver enormously enlarged, and pressing up the diaphragm, so as to come in contact with the heart—thus accounting for the pulsation in epigastrio and in the right hypochondrium.

---

* Mr. Ward. Med. and Phys. Journ. No. 291.

